# Imprint of preterm birth with very low birth weight on optic disc OCT in adulthood—A two‐country birth cohort study

**DOI:** 10.1111/aos.16771

**Published:** 2024-10-17

**Authors:** Maarit K. Kulmala, Anna Jørgensen, Dordi Austeng, Kari Anne I. Evensen, Eero Kajantie, Tora Sund Morken, Anna Majander

**Affiliations:** ^1^ Population Health Unit Finnish Institute for Health and Welfare Helsinki Finland; ^2^ Department of Ophthalmology, Helsinki University Hospital University of Helsinki Helsinki Finland; ^3^ Department of Neuromedicine and Movement Science Norwegian University of Science and Technology (NTNU) Trondheim Norway; ^4^ Department of Ophthalmology, St. Olavs Hospital Trondheim University Hospital Trondheim Norway; ^5^ Department of Clinical and Molecular Medicine NTNU Trondheim Norway; ^6^ Children's Clinic St. Olavs Hospital, Trondheim University Hospital Trondheim Norway; ^7^ Department of Rehabilitation Science and Health Technology Oslo Metropolitan University Oslo Norway; ^8^ Clinical Medicine Research Unit, MRC Oulu Oulu University Hospital and University of Oulu Oulu Finland; ^9^ Helsinki Children’s Hospital University of Helsinki and Helsinki University Central Hospital Helsinki Finland

**Keywords:** adult, optical coherence tomography (OCT), preterm, very low birth weight

## Abstract

**Purpose:**

To determine the pattern, degree and prevalence of optic disc optical coherence tomography (OCT) alterations in adults born preterm with very low birth weight (VLBW; birth weight < 1500 g).

**Methods:**

Optic disc OCT was assessed in 98 VLBW participants and 139 term‐born controls from birth cohorts in Finland and Norway at the mean age of 36 years. The participants had not been treated for retinopathy of prematurity and had no diagnosed brain abnormality. OCT assessment included parameters for optic disc size, neural rim and peripapillary retinal nerve fibre layer thickness (pRNFLT), and for the foveal developmental stage. Background data, visual acuity, refractive error and intraocular pressure were recorded.

**Results:**

In the VLBW group, optic disc neural rim and pRNFLT were significantly decreased, most frequently in the nasal and inferior sectors. In 40% (95% CI: 33–47) of the VLBW eyes, nerve fibre thickness of at least one optic disc sector was below the fifth percentile of the control group, including 9% (95% CI: 6–14) subgroup falling below the first percentile, that is, clinically below normal limits. VLBW participants with nerve fibre thickness below the fifth percentile did not differ by perinatal data or foveal developmental stage from the other VLBW participants. All participants had normal visual acuity.

**Conclusion:**

A moderate decrease of the optic disc neural rim and pRNFLT is frequently seen in clinically healthy adults born preterm with VLBW. Awareness of the VLBW‐related optic disc nerve fibre shallowness especially in the inferior and nasal sectors is important while evaluating acquired optic disc pathology in adulthood.

## 1 INTRODUCTION

Being born preterm with very low birth weight (VLBW; <1500 g) disrupts a complex developmental period of eye maturation that includes both neuronal proliferation and apoptosis (Shen et al., 2022). The optic nerve axonal count peaks in the second trimester and decreases until approximately the 32nd gestational week, followed by the optic disc size reaching 75% of its adult dimensions at full‐term birth (Provis et al., 1985; Rimmer et al., 1993; Thomas et al., 2017). Optic disc alterations, such as an enlarged cup area, volume, cup/disc area ratio and horizontal cup/disc ratio, or reduced rim area, have frequently been reported in children born preterm and having additional complications in the central nervous system (CNS) or a history of retinopathy of prematurity (ROP), central visual impairment but also without any underlying factors other than prematurity (Åkerblom et al., 2012; Al‐Abaiji et al., 2024; Fieß et al., 2024; Fledelius, 1978; Ruberto et al., 2014; Wang et al., 2012a; Wenner et al., 2023). Gestational weeks at preterm birth can have an impact on the optic disc morphology, but birth weight has been shown as an independent predictor of peripapillary retinal nerve fibre layer thickness (pRNFLT) (Raffa et al., 2021; Shen et al., 2022). The macula of preterm born is characterized by a specific developmental abnormality later in life with increased retinal thickness in the foveal center, suggested to have a common developmental origin particularly with the temporal sector pRNFLT (Ecsedy et al., 2007; Fieß, Schäffler, et al., 2022; Hammer et al., 2008; Kuruvilla et al., 2018; Recchia & Recchia, 2007; Wang et al., 2012a). The optic disc morphology of an individual born preterm with VLBW is, thus, potentially affected by multiple parallel and background factors.

Optical coherence tomography (OCT) of the optic disc belongs to the key diagnostics of optic nerve diseases, including glaucoma, and can also reflect various CNS disorders (Gür Güngör & Ahmet, 2017; Hong et al., 2018; Jacobson et al., 1997; McLoone et al., 2006; Scheiman, 1984). Furthermore, pRNFLT decreases with age without any associated pathology (Coleman‐Belin et al., 2023). With regard to the diagnosis of acquired optic nerve diseases, recognition of congenital alterations, such as the impact of birth history, is important. The evaluation of the long‐term effects of prematurity on the optic disc OCT morphology has become timely, as the first generation of preterm‐born infants receiving neonatal intensive care are entering middle age, whereby also the risk of degenerative diseases appearing with aging and requiring diagnostic examinations increases. Although altered optic disc features have long been recognized in infants and children born preterm, data from OCT characterization in adulthood are limited (Fieß, Schäffler, et al., 2022; Pétursdóttir et al., 2024). Applying the optic disc findings reported in children to adulthood is also complicated by variations of the prematurity‐related confounding variables in the studied series.

In this study of two Nordic birth cohorts, we assess optic disc OCT features of 98 adult participants born preterm with VLBW and 139 term‐born controls. The optic disc OCT alterations are evaluated in relation to birth characteristics, developmental stage of the fovea and a few OCT indexes used in the glaucoma diagnostics. The aim is to determine the pattern, degree and prevalence of optic disc OCT alterations related to preterm birth with VLBW in a cohort of clinically healthy individuals with normal visual acuity and without a history of treated ROP or diagnosed CNS abnormality. Our hypothesis is that preterm birth with VLBW modifies optic disc morphology also in the absence of verified ocular or CNS complications of prematurity.

## 2 MATERIALS AND METHODS

### 2.1 Study design and participants

Participants were recruited and background data recorded from two longitudinal follow‐up birth cohorts: the Helsinki Study of Very Low Birth Weight Adults (HeSVA cohort; born in 1978–1985) and the Norwegian University of Science and Technology – Low Birth Weight in a Lifetime Perspective study (NTNU LBW Life cohort; participants born in 1986–1988) as described elsewhere by the present authors, including a detailed non‐participant analysis (Jussinniemi et al., 2024; Kulmala et al., 2024). A singleton term born infant of the same sex and not born small for gestational age (SGA), group‐matched for sex, age and birth hospital was selected for each VLBW at the beginning of the HeSVA cohort. The NTNU LBW Life cohort comprised VLBW infants admitted to the neonatal care unit at St. Olavs Hospital, Trondheim, Norway, and non‐SGA control participants born at term. Previous young adulthood assessments were completed during 2004–2005 in HeSVA at a mean age of 22.5 years and in NTNU LBW Life during 2013–2014 at a mean age of 26.4 years. All participants, who had not denied further contact, were invited to the current study.

Altogether 274 participants underwent ophthalmological examination (175 of HeSVA and 99 of NTNU LBW Life) with a protocol modified from the Adults Born Preterm International Collaboration recommendation for follow‐up studies of adults born preterm (Kajantie et al., 2021). Ophthalmological examination of both eyes was performed in parallel at both study sites (Helsinki and Trondheim) between September 2019 and December 2020, and included assessment of optic disc and macular OCT, best‐corrected visual acuity (BCVA) using the Early Treatment of Diabetic Retinopathy Study (ETDRS) visual acuity testing protocol (Brown et al., 2006) and intraocular pressure (IOP) measured with a rebound tonometer (iCare, Icare Oy, Vantaa, Finland).

**FIGURE 1:**
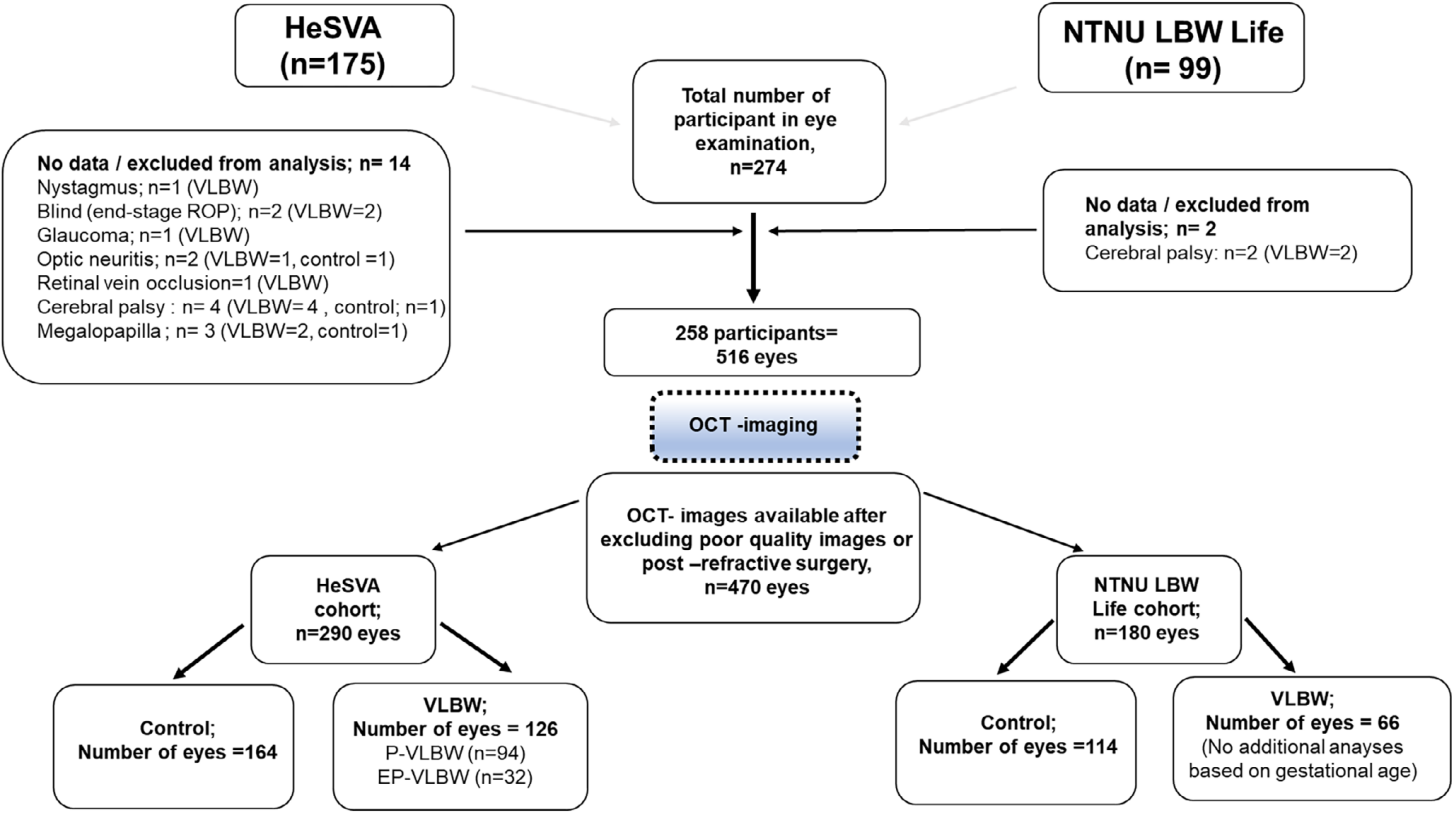
Flow diagram of study participants in the Helsinki Study of Very Low Birth Weight Adults (HeSVA) and the Norwegian University of Science and Technology – Low Birth Weight in a Lifetime Perspective study (NTNU LBW Life). HeSVA cohort included infants born 1978–1985 in Helsinki University Central Hospital, Helsinki, Finland. Eye examination was conducted for 175 of them in a study visit in 2019–2020, and optic disc optical coherence tomography images of 146 participants (including 64 VLBW and 82 controls) were available for the analyses. NTNU LBW life cohort included infants born 1986–1988 in St.Olavs Hospital, Trondheim University Hospital, Trondheim, Norway. Eye examination was conducted for 99 of them in a study visit in 2019–2020, and optic disc optical coherence tomography images of 90 participants (33 VLBW and 57 controls) were available for the analyses. HeSVA, the Helsinki Study of Very Low Birth Weight Adults; NTNU LBW Life, the Norwegian University of Science and Technology – Low Birth Weight in a Lifetime Perspective study; OCT, optical coherence tomography; ROP, retinopathy of prematurity; VLBW, very low birth weight.

Sixteen participants were excluded from the current study due to a diagnosis of glaucoma (post‐operative and secondary), optic neuritis, cerebral palsy, optic atrophy caused by retinal vein occlusion, megalopapilla (optic disc area >3 mm^2^) or insufficient fixation for the optic disc OCT imaging due to nystagmus, very low visual acuity or lack of co‐operation, as detailed in the flow‐chart in Figure 1. Optic disc OCT data of 470 eyes of 237 participants (two participants had only one eye included) were available for the analyses, after the exclusion of images with poor quality (20 eyes of 10 participants) and of the eyes that had previously undergone refractive surgery (26 eyes of 13 participants). None of the participants had been treated for ROP, but at the time the participants were born, the screening programs for retinopathy of prematurity (ROP) were not yet in place in Norway and Finland, and systematic data on ROP were not available in medical records. VLBW participants with diagnosed cerebral palsy (CP) were also excluded from the analyses, since CP may be associated with specific changes in optic disc morphology (Ghate et al., 2016). Optic disc OCT measurements and background data of VLBW with CP (*n* = 4) are presented in Supporting Information 1. An original diagnosis of CP was mainly based on clinical findings since brain screening by magnetic resonance imaging (MRI) was not yet available for premature babies at the time of birth of the participants. For the assessment of the potential impact of extremely preterm birth on optic disc OCT, VLBW participants of the HeSVA cohort were additionally stratified into subgroups of extremely preterm (EP‐VLBW, born before completed 28 gestational weeks, *n* = 17) and preterm (P‐VLBW, born at completed 28 gestational weeks or more, *n* = 49), since extremely preterm birth has been associated with higher risks for developmental complications also in the eye compared to birth at a later gestational age (Darlow et al., 2018; Hellgren et al., 2016; Jain et al., 2022; Lindqvist et al., 2007; Marlow et al., 2005; Pétursdóttir et al., 2020). The small number of participants in the NTNU cohort did not justify analyses between subgroups divided by gestational weeks. The distribution of participants in the cohorts and study groups are presented in Figure 1. Altogether 192 eligible eyes of VLBW participants (HeSVA: *n* = 126; NTNU LBW Life: *n* = 66) and 278 eyes of control participants (HeSVA: *n* = 164; NTNU LBW Life: *n* = 114) were included in the study.

### 2.2 Optic disc OCT imaging

In the two study sites, optic disc OCT imaging was performed with the available OCT devices that used different strategies for pRNFLT assessment, and data from both study sites were analysed separately including site‐specific controls.

In the HeSVA cohort, optic disc OCT was scanned using the Spectralis FD‐OCT (ver. 6.12.4.0, Glaucoma module, Heidelberg Engineering Carlsbad, CA, USA) and the Anatomic Positioning System (APS) utilizing eye‐tracking for referring to the papillo‐macular axis and including adjustment for fovea to disc angle. The Bruch's membrane opening (BMO) area and the minimum rim width (MRW), corresponding to the minimum distance between the edge of the Bruch's membrane opening and the internal limiting membrane around the optic nerve head in 24 radial scans centered on the BMO, were recorded by the software algorithm. The global average and the average MRW for each six sectors (inferotemporal, inferonasal, nasal, superonasal, superotemporal and temporal) were included in the data analysis. The pRNFLT was obtained from a 3.5‐mm diameter circular scan centered on the BMO and recorded as the global average and the average thickness for each of the six optic disc sectors. Retinal structures, automatically identified by the software algorithm, were reviewed by MK, and adjusted in case of misplacement.

Optic disc imaging of the NTNU LBW Life cohort was performed using Cirrus High‐Definition OCT 5000/500 (HD‐OCT, Carl Zeiss Meditec, Dublin, CA, USA). Values for the optic disc and rim areas, pRNFLT for four sectors (temporal, inferior, nasal and superior) and global pRNFLT were derived from Optic Disc Cube 200 × 200 scans. The software automatically interpolates pRNFLT values from the volume scans using a reference plane 200 μm above the retinal pigment epithelium at a 3.46 mm diameter circle centered in the optic disc. Retinal layer segmentation lines, automatically recorded by the software, were inspected, and manually adjusted by AJ in cases of error.

### 2.3 Optic disc OCT glaucoma indexes

In the HeSVA cohort, the inferior/superior (IS) pRNFLT ratio was calculated for the pRNFLT and MRW using combined mean inferotemporal and inferonasal sector thicknesses divided by the combined mean superotemporal and superonasal values, respectively (Law et al., 2016; Park et al., 2018). In addition, a global pRNFLT/MRW ratio was calculated for the HeSVA cohort (Boussion et al., 2023). In the NTNU LBW Life cohort, the IS pRNFLT ratio was calculated for each eye using the corresponding mean sectoral values.

### 2.4 Macular developmental arrest index

In both study sites, macular OCT was imaged using SD‐OCT (Heidelberg Engineering, Heidelberg, Germany). Retinal layers were automatically segmented, and mean thickness values of the individual retinal layers were calculated by the Heidelberg Eye Explorer Software tool (HEYEX, version 2.5.1, Heidelberg Engineering, Heidelberg, Germany) (Jørgensen et al., 2024). In addition, The ETDRS grid placement and segmentation of individual retinal layers in each single B‐scan were checked and manually corrected if they were misplaced. The thickness of the outer nuclear layer (ONL) and a combined thickness of the inner retinal layers (IRL) including retinal nerve fibre, ganglion cell, inner plexiform, inner nuclear and outer plexiform layers were recorded at the foveolar center defined as the deepest point of foveal depression. The macular developmental arrest (MDA) index, defined as the ONL thickness divided by the IRL thickness (the ONL/IRL‐ratio), was calculated manually for each participant (Bowl et al., 2018).

### 2.5 Statistical analyses

Since HeSVA and NTNU LBW Life study sites used different OCT platforms and scanning strategies, the cohorts were analysed separately. Statistical analyses were performed using the SPSS software 28.0.0.0(190) (IBM SPSS Statistics, NY, USA). Normality was assessed via visual inspection of histograms and Q–Q plots of residuals and was valid for all parameters except the MDA index showed significant skewness and was therefore converted to logarithmic values for analyses. Continuous background data were compared between the groups using Student's t‐test for normally distributed continuous data and Mann–Whitney *U*‐test for non‐parametric variables. Chi‐Square test was used for prevalence (percentages) comparisons of the background factors between the study groups. A mixed model (generalized linear) was used for comparisons of the ocular data between the groups. Data from both eyes were used in the analyses, with participants entered as a random effect in the model to account for potential correlations between the two eyes. Mixed models included adjustment for age and sex in comparisons of all ocular data between the groups. OCT data and IOP were also adjusted for the spherical equivalent of the refractive error that is known to affect both OCT and IOP readings directly, or indirectly as a reflection of axial length, at least in higher‐order refractive errors. The rim area, MRW and pRNFLT comparisons were additionally adjusted also for the optic disc or BMO area. *p*‐values <0.05 were considered statistically significant. Correlations were tested using Pearson's or Spearman's test. Multiple comparisons were adjusted using Benjamini–Hochberg correction for *p* values.

In analogy to the grading of normality of the pRNFLT values provided by the SD‐OCT and HD‐OCT devices, the fifth (P_5_) and first (P_1_) percentiles of the pRNFLT values were calculated for the control group in both cohorts, corresponding to the borderline (yellow; the fifth percentile) and abnormal (red; the first percentile) colour‐coded zones in both OCT platforms. The same percentiles of the MRW data were calculated for the HeSVA control group. The eyes of the VLBW group presenting with pRNFLT, or MRW, below the fifth (P_5_) and first percentiles (P_1_) of the corresponding study‐sites control group (HeSVA and NTNU LBW Life separately) in at least one optic disc sector were recorded and characterized as a subgroup with borderline (VLBW < P_5_) or abnormal (VLBW < P_1_) optic disc OCT, respectively. The participant subgroup VLBW < P_5_ (in Table 1) included participants with sectoral pRNFLT or MRW below the fifth percentile at least in one eye. Data of the VLBW < P_5_ subgroup were compared to the control data and to the VLBW group with pRNFLT, or MRW, of the level of the fifth control percentile or better (VLBW ≥ P_5_), depending on the setting, and using mixed models as described above.

**TABLE 1:** Background characteristics of the HeSVA and NTNU LBW Life cohorts.

(a) HeSVA cohort	Control	VLBW		VLBW ≥ P_5_	VLBW < P_5_	
Participants, *n* (%)	82	65		32	33 (51%)	
	Mean (SD) or *n* (%)		*p*	Mean (SD) or *n* (%)		*p* *
*Perinatal data*						
Gestational age (weeks)	40.2 (1.2)	29.5 (2.4)	**<0.001**	29.8 (2.4)	29.3 (2.4)	0.337
Extremely preterm birth (<28th week), *n* (%)	0	17 (26%)		8 (25%)	9 (27%)	0.835
Birth weight (g)	3612 (489)	1152 (207)	**<0.001**	1197 (193)	1109 (214)	0.089
Birth weight as SD score	0.1 (1.0)	−1.4 (1.5)	**<0.001**	−1.4 (1.5)	−1.4 (1.5)	0.700
Small for gestational age, *n* (%)	0	37 (57%)	**<0.001**	15 (47%)	18 (55%)	0.585
Birth length (cm)	50.3 (1.9)	37.1 (2.3)	**<0.001**	37.4 (2.2)	36.8 (2.4)	0.365
Apgar score 1 min	8.7 (0.8)	6.0 (2.4)	**<0.001**	6.3 (2.2)	5.9 (2.6)	0.654
Apgar score 5 min	9.4 (0.5)	7.3 (1.9)	**<0.001**	7.1 (1.9)	7.4 (1.6)	0.620
Sepsis, yes	0	4		1	3	
Seizures, yes	0	2		0	2	
Bronchopulmonary dysplasia >28 days, *n* (%)	0	16 (25%)		5 (16%)	11 (33%)	0.056
Maternal age (years)	29.9 (5.3)	30.4 (4.4)	0.147	30.9 (5.3)	29.9 (3.4)	0.392
*Participant data*						
Age at follow‐up (years)	38.0 (2.3)	37.9 (2.2)	0.632	37.8 (2.1)	38.1 (2.3)	0.663
Female, *n* (%)	47 (57%)	37 (57%)	0.962	22 (69%)	15 (45%)	0.058

(b) NTNU LBW life cohort	Control	VLBW		VLBW ≥ P_5_	VLBW < P_5_	
Participants, *n* (%)	57	33		20	13 (39%)	
	Mean (SD) or *n* (%)		*p*	Mean (SD) or *n* (%)		*p* *
*Perinatal data*						
Gestational age (weeks)	39.8 (1.2)	30.1 (2.5)	**<0.001**	30.1 (2.9)	30.2 (1.8)	0.953
Extremely preterm birth (<28th week), *n* (%)	0	6 (18%)		2 (15%)	4 (20%)	0.737
Birth weight (g)	3682 (479)	1294 (180)	**<0.001**	1317 (159)	1260 (211)	0.387
Birth weight as SD score	0.3 (1,0)	−1,0 (2.0)	**<0.001**	−0.7 (2.2)	−1.3 (1.7)	0.881
Small for gestational age, (%)	0	15 (45%)		9 (45%)	6 (46%)	0.948
Birth length (cm)	51.1 (1.9)	39.2 (3.9)	**<0.001**	38.7 (4.3)	40.3 (2.5)	0.305
Apgar score 1 min	8.9 (0.4)	7.1 (2.0)	**<0.001**	7.9 (1.3)	5.8 (2.4)	**0.009** **
Apgar score 5 min	9.7 (1.3)	9.0 (0.9)	**<0.001**	9.1 (0.9)	8.8 (1.0)	0.310
Sepsis, yes	0	2		0	2	
Seizures, yes	0	1		0	1	
Bronchopulmonary dysplasia >28 days, *n*	0	3		1	2	
Maternal age (years)	30.9 (4.4)	30.1 (4.8)	0.213	30.5 (5.2)	29.4 (4.2)	0.522
*Participant data*						
Age at follow‐up (years)	32.6 (0.5)	32.4 (0.8)	0.051	32.4 (0.8)	32.3 (0.7)	0.768
Female, *n* (%)	31 (54%)	21 (64%)	0.507	12 (60%)	9 (69%)	0.737

*Note*: (a) Comparison between the VLBW and control groups in both cohorts, and between the VLBW participants with all sectoral optic disc neural rim and peripapillary nerve fibre layer thicknesses at least the 5th percentile of the control group (VLBW ≥ P_5_) and VLBW participants with at least one sectoral thickness below the 5th percentile of the control group (VLBW < P_5_). (b) Comparisons were performed using Student's *t*‐test for the normally distributed continuous data and Mann–Whitney *U*‐test for non‐parametric variables. Chi‐Square test was used for prevalence (percentages) comparisons of the background factors between the study groups.

Abbreviations: HeSVA, the Helsinki Study of Very Low Birth Weight Adults; NTNU LBW Life, the University of Science and Technology – Low Birth Weight in a Lifetime Perspective study; OCT, optical coherence tomography, P_1_, 1st percentile; P_5_, 5th percentile; SD, standard deviation; VLBW, very low birth weight.

* Comparison between the VLBW ≥ P_5_ and VLBW < P_5_ groups.

** *p* value = 0.054 for NTNU LBW Life Apgar score 1 min, after adjustment for multiple comparisons using Benjamini–Hochberg correction for *p* values (For other variables, the significance did not change after adjustment.).

Significant P values below 〈0.05 bolded.

### 2.6 Ethics

The study followed the guidelines of the Declaration of Helsinki and was approved by the Regional Committee for Medical Research Ethics in Central‐Norway (REK/23879) and by the Ethics Committee IV of Helsinki University Hospital (HUS/1157/2019) in Finland. The study had institutional approval by St. Olavs Hospital, Trondheim (Norway) and Helsinki University Hospital, Helsinki (Finland). Written informed consent was obtained from all participants.

## 3 RESULTS

Background characteristics of the VLBW and control participants differed only regarding the perinatal data, whereas participant characteristics at the time of examination were similar in the VLBW and control groups, Table 1. The foveal developmental stage assessed by the MDA index was significantly lower in the VLBW group compared to control eyes, including 38% of the VLBW eyes with an MDA index below the fifth percentile of the controls, Table 2. ETDRS score was a few letters lower in the VLBW group compared to controls, but still above the normal ETRDS score of 80 (Elflein et al., 2015), but after adjustment with the Benjamini–Hochberg method the difference did not remain significant. IOP or spherical equivalent of the refractive error did not differ between the VLBW and control participants.

**TABLE 2:** Ocular characteristics of the HeSVA and NTNU LBW Life cohorts.

	Control	VLBW	*p*	VLBW ≥ P_5_	VLBW < P_5_	*p* *
	Mean (SD) [95% CI]					
*HeSVA*						
Number of eyes (%)	164	126		73	53 (42%)	
MDA index^a^	6.6 (4.0) [6.0, 7.2]	3.9 (2.5) [3.4, 4.3]	**<0.001**	4.0 (2.3) [3.4, 4.5]	3.7 (2.7) [3.0, 4.6]	0.717
ETDRS score^a^	90.5 (4.3) [89.9, 91,2]	88.7 (5.9) [87.7, 89.8]	**0.021** **	89.0 (6.2) [87.5 90.4]	88.4 (5.4) [86.9, 89.9]	0.478
Spherical equivalent (D)^a^	−1.29 (2.17) [−1.94, −2.67]	−1.62 (2.38) [−2.04, −1.2]	0.358	−1.5 (2.30) [−2.02, −0.97]	−1.94 (2.55) [−2.50, −1.09]	0.713
IOP (mmHg)^b^	14.3 (3.7) [13.7, 14.9]	14.8 (3.4) [14.2, 15.4)	0.372	14.3 (2.8) [13.6, 14.9]	15.3 (4.0) [14.4, 16.6]	0.612
BMO area (mm^2^)^b^	2.01 (0.41) [1.94, 2.07]	2.00 (0.44) [1.92, 2.08]	0.965	2.05 (0.43) [1.94, 2.15]	1.94 (0.45) [1.81, 2.06]	0.511
I/S pRNFLT ratio^c^	1.07 (0.13) [1.05, 1.09]	1.02 (0.19) [1.00, 1.06]	0.058	1.00 (0.12) [0.98, 1.03]	1.05 (0.26) [0.98, 1.13]	0.305
I/S MRW ratio^c^	1.14 (0.14) [1.12, 1.17]	1.10 (0.16) [1.07, 1.12]	**0.047** **	1.08 (0.14) [1.04, 1.11]	1.12 (0.18) [1.07, 1.17]	0.380
gRNFLT/MRW ratio^c^	0.29 (0.52) [0.27, 0.29]	0.28 (0.06) [0.27, 0.29]	0.825	0.28 (0.04) [0.27, 0.29]	0.28 (0.07) [0.27, 0.30]	0.677
*NTNU LBW life*						
Number of eyes (%)	114	66		42	24 (36%)	
MDA index^a^	6.6 (2.9) [6.1, 7.2]	4.6 (2.4) [3.9, 5.2]	**<0.001**	4.3 (2.5) [3.5, 5.2]	5.0 (2.2) [3.8, 6.1]	0.320
ETDRS score^a^	86.6 (4.2) [85.8, 87.4]	84.8 (5.0) [83.6, 86.1]	0.052	85.7 [4.1) [84.8, 86.9]	83.3 (6.2) [80.7, 86.0]	**0.026** **
Spherical equivalent (D)^a^	−1.10 (2.15) [−1.50, −0.70]	−0.48 (1.93) [−0.95, 0.01]	0.216	−0.35 (−0.25) [−0.99, 0.29]	−0.71 (1.71) [−1.43, 0.14]	0.856
IOP (mmHg)^b^	13.3 (2.3) [12.8, 13.8]	14.8 (3.1) [13.9, 15.7]	0.192	16.2 (3.6) [14.3, 18.1]	14.2 (2.7) [13.2, 15.2]	0.367
Disc area (mm^2^)^b^	1.73 (0.30) [1.73, 1.84]	1.83 (0.27) [1.76, 1.89]	0.742	1.83 (0.29) [1.74, 1.92]	1.81 (0.24) [1.71, 1.92]	0.795
I/S pRNFLT ratio^d^	1.11 (0.20) [1.07, 1.15]	1.11 (0.15) [1.07, 1.15]	0.700	1.11 (0.14) [1.07, 1.15]	1.10 (0.16) [1.07, 1.17]	0.467

*Note*: Comparison between the VLBW and control groups in both cohorts, and between the VLBW participants with all sectoral optic disc neural rim and peripapillary nerve fibre layer thicknesses at least the 5th percentile of the control group (VLBW ≥ P_5_) and VLBW participants with at least one sectoral thickness below the fifth percentile (VLBW < P_5_). Comparisons were performed using mixed models with a participant set as a random effect and adjusted for the following variables: ^a^age, sex; ^b^age, sex, spherical equivalent; ^c^age, sex, spherical equivalent, BMO area; ^d^age, sex, spherical equivalent, disc area.

Abbreviations: BMO, basement membrane opening; CI, confidence interval; D, diopter; ETDRS, Early Treatment of Diabetic Retinopathy Study; gRNFLT/MRW ratio, ratio of the global retinal nerve fibre layer thickness to the global minimum rim width; HeSVA, the Helsinki Study of Very Low Birth Weight Adults; IOP, intraocular pressure; I/S MRW ratio, ratio of the minimum rim width of the inferior sector to the width of the superior sector; I/S pRNFLT ratio, ratio of the retinal nerve fibre layer thickness of the inferior sector to the thickness of the superior sector; MDA, macular developmental arrest; NTNU LBW Life, the Norwegian University of Science and Technology—Low Birth Weight in a Lifetime Perspective study; SD, standard deviation; VLBW, very low birth weight.

* Comparison between the VLBW ≥ P_5_ and VLBW < P_5_ eyes.

** *p* values not significant after adjustment for multivariate comparison by the Benjamini–Hochberg method.

Significant P values below <0.05 bolded.

**FIGURE 2:**
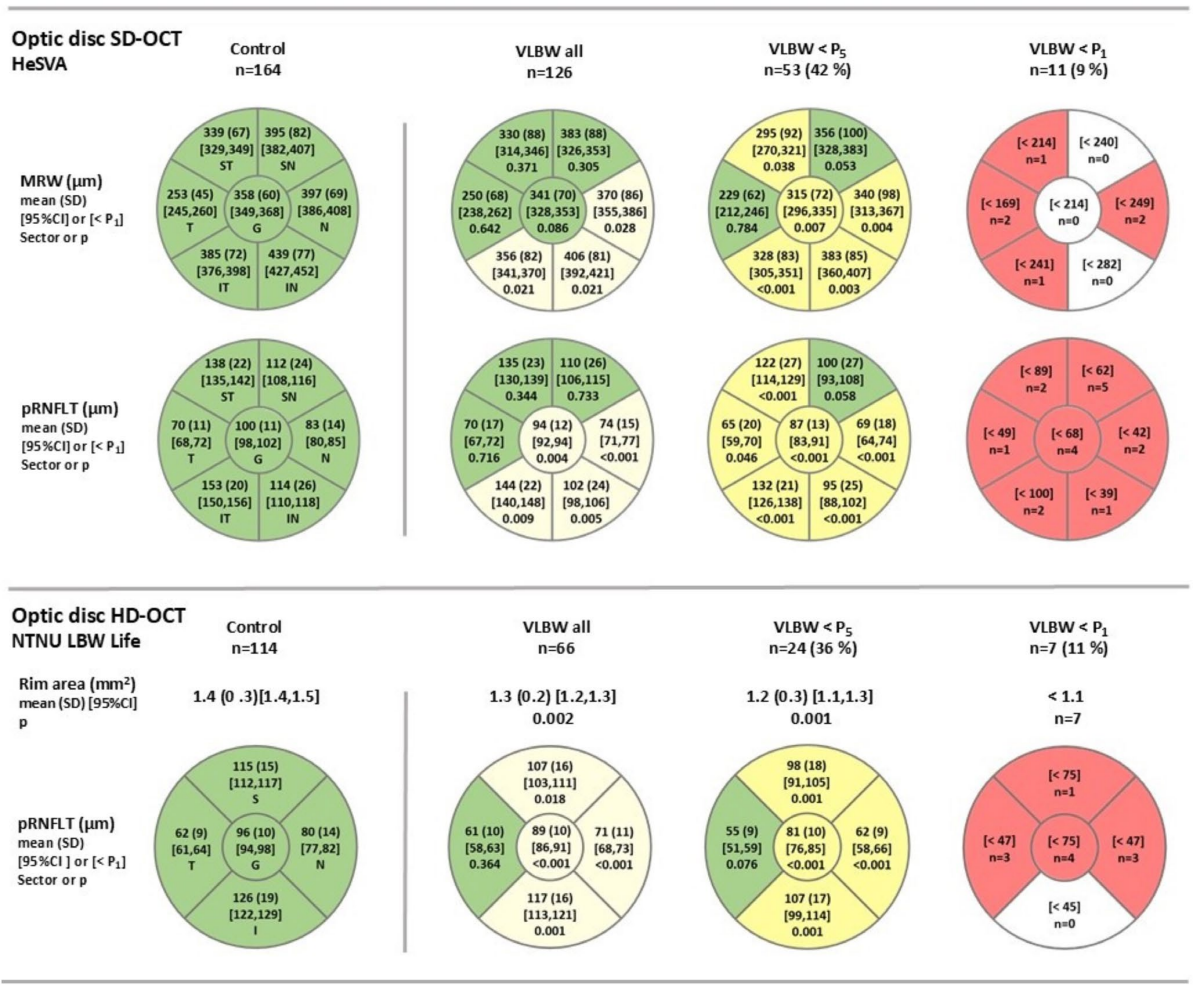
Optic disc OCT data of adults born preterm with very low birth weight (VLBW) and term‐born controls of HeSVA and NTNU LBW Life cohorts, assessed by Spectral domain (SD‐OCT) and Cirrus high‐definition (HD‐OCT) OCT platforms, respectively, and including sectoral distribution of the nerve fibre thicknesses and stratification into VLBW subgroups based on the severity of deviation in comparison to controls. Abbreviations: CI, confidence interval; HeSVA, the Helsinki Study of Very Low Birth Weight Adults; I, inferior optic disc sector; IN, inferonasal optic disc sector; IT, inferotemporal optic disc sector; MRW, minimum rim width; N, nasal optic disc sector; NTNU LBW Life, the Norwegian University of Science and Technology—Low Birth Weight in a Lifetime Perspective study; OCT, optical coherence tomography; pRNFLT, peripapillary nerve fibre layer thickness; S, superior optic disc sector; SD, standard deviation; SN, superonasal optic disc sector; ST, superotemporal optic disc sector; T, temporal optic disc sector; VLBW < P_1_, below the first percentile of the control; VLBW < P_5_, below the fifth percentile of the control. Additional information: In the HeSVA cohort, MRW and pRNFLT were assessed globally and in six sectors. In the NTNU LBW Life cohort, OCT assessment of optic disc rim area and pRNFLT in four sectors and globally are presented. VLBW eyes with MRW or pRNFLT below the fifth (VLBW < P_5_) and the first (VLBW < P_1_) percentiles of the control at least in one optic disc sector are presented as subgroups including the percentage of these eyes in the VLBW group in each birth cohort. For the VLBW < P_1_ group, the threshold values of MRW or pRNFLT for the first control percentile are presented [< P_1_]. *p* values refer to comparisons between VLBW and control groups within each birth cohort, using mixed models with participants entered as a random effect and including adjustments for age, sex, spherical equivalent of the refractive error and BMO area in the HeSVA cohort, and optic disc area in the NTNU LWB Life cohort. Multiple comparisons were adjusted using Benjamini–Hochberg correction for *p* values.

Optic disc size, assessed by the BMO and optic disc areas in the two birth cohorts, did not differ between the VLBW and control groups, Table 2. Optic disc OCT data, including sectoral distribution of nerve fibre thicknesses and subgroup data stratified based on the degree of alterations in the VLBW eyes compared to controls, are presented in Figure 2. The optic disc neural rim and peripapillary nerve fibre layer were thinner in the VLBW participants compared to controls, as indicated by the optic disc rim area, the MRW and the pRNFLT. In the eyes of the VLBW participants of both cohorts, the pRNFLT of the temporal sector was within the control range, but that of the nasal and inferior sectors were reduced compared to controls. In the NTNU LBW Life cohort assessed by Cirrus HD‐OCT, also the superior sector pRNFLT was thinner than in the control group.

Forty‐two percent (95% CI: 33–51) of eyes in the HeSVA VLBW group had pRNFLT or MRW below the fifth percentile of the control group (VLBW < P_5_) at least in one optic disc sector, referring to borderline nerve fibre thickness. In the NTNU LBW Life cohort, a borderline (P_5_) sectoral pRNFLT was found in 36% (95% CI: 25–49) in VLBW, that is, within a similar range to that in the HeSVA cohort. The percentage of VLBW participants with borderline (P_5_) nerve fibre thickness at least in one eye was 51% [95% CI: 38–63] in HeSVA and 39% [95% CI 23–58] in NTNU LBW Life. Along with a more pronounced reduction of nerve fibre thickness compared to controls, also the superonasal and temporal sectors were involved in the eyes of the VLBW in the HeSVA cohort with borderline (P_5_) pRNFLT or MRW. In the NTNU LBW Life cohort, the sectoral distribution of pRNFLT alterations of the VLBW eyes were similar independent of the degree of the deviation and involved all sectors except temporal. Nine percent of eyes (95% CI: 4–15) in HeSVA VLBW, and 11% (95% CI: 4–21) in NTNU LBW Life, respectively, had MRW or pRNFLT below the first percentile (P_1_) of the control, consistent with abnormal optic disc OCT. Unlike optic discs with a borderline degree of MRW or pRNFLT thicknesses compared to control, nerve fibre loss in the most severely affected discs covered all sectors without any specific distribution pattern.

In both cohorts, comparisons between VLBW participants with nerve fibre thickness below and at least the fifth percentile (P_5_) showed similar MDA index, IOP, optic disc size, gestational age and birth weight, as well as the proportion of participants born extremely preterm or SGA, Tables 1 and 2. The only difference was a lower Apgar score at 1 min in the NTNU LBW Life cohort (*p* = 0.009), however, it was not significant after adjustment for multiple comparisons using Benjamini–Hochberg correction (*p* = 0.054). Within the VLBW of both cohorts, the ETDRS letter score or MDA index did not show a significant correlation with any of the optic disc OCT parameters, including temporal sector RNFLT. In the five eyes of the HeSVA cohort with temporal sector pRNFLT below the fifth percentile (P_5_) of the control, the MDA index was very low (mean 2.2, range 1.5–3.8) referring to abnormally thick fovea.

The only difference in comparisons of the optic disc OCT indexes used in the diagnosis of glaucoma was found in the I/S MRW ratio between the VLBW and control group in the HeSVA cohort, but not between the VLBW < P_5_ and VLBW ≥ P_5_ groups. The I/S gRNFLT ratios were similar in all studied eyes of both cohorts, and comparable also in the two study sites, Table 2. The gRNFLT/MRW ratio available in the HeSVA cohort was also similar in the studied eyes, and independent of the degree of optic disc OCT alterations.

Evaluation of the impact of extremely preterm birth on optic disc OCT in the HeSVA cohort showed a slightly larger BMO area in the EP‐VLBW group (mean 2.10 mm^2^ [95% CI: 1.94–2.27]), compared to P‐VLBW group (mean 1.96 mm^2^ [95% CI:1.88–2.05], *p* = 0.426). Even after adjustment for the BMO area, participants in the EP‐VLBW group had slightly (*p* = 0.512) lower MRW (global 316 μm, SD = 41, 95% CI: 301–333) than participants in the P‐VLBW group (global MRW 341 μm, SD = 77, 95% CI: 326–356), whereas pRNFLT was similar independent of the gestational age. The prevalence of nerve fibre thickness below the fifth control percentile was also similar in the EP‐VLBW and P‐VLBW eyes, 40% (CI: 24%–59%) versus 43% (95% CI: 33%–53%). The degree of foveal maturation assessed by the MDA index was lower in the EP‐VLBW group compared to the P‐VLBW group (mean 3.0 [95% CI: 2.5–3.6] vs. 4.2 [95% CI: 3.6–7.3], *p* = 0.152), and both had significantly lower index compared to controls (6.6 [95% CI: 6.0–7.2], *p* < 0.001). However, no correlation could be shown between the MDA index, or glaucoma indexes, and the optic disc OCT data.

## 4 DISCUSSION

This optic disc OCT study of 470 eyes comparing the VLBW and control groups showed that a moderate thinning of the neuroretinal rim and peripapillary nerve fibre layer is frequently seen in adults born preterm with VLBW. Importantly, these alterations were recorded in clinically healthy individuals with normal level visual acuity, who did not have confounding background variables, such as a previously treated ROP or diagnosed CNS abnormality, other than preterm birth with VLBW.

This study was performed in two birth cohorts with similar inclusion criteria and participant characteristics of both the VLBW and control groups. Optic disc OCT imaging was, however, performed with two different platforms that use different algorithms for the pRNFLT assessment and sectoral segregation. Despite these methodological differences, the degree and prevalence of the global pRNFLT loss were similar in both VLBW birth cohorts compared to the controls of the same cohort. Consistent findings in two cohorts studied with different OCT platforms indeed strengthen the validity of our results.

The VLBW‐related optic disc OCT alterations showed a specific pattern of sectoral distribution. Preservation of the optic disc nerve fibres in the temporal sector, but reduced thickness in the nasal and inferior sectors, was a common feature for the VLBW group. In the VLBW cohort examined by Cirrus HD‐OCT, the superior sector pRNFLT was also reduced, while in the VLBW group imaged on the SD‐OCT platform, the superior sectors differed from the control only in eyes with more pronounced nerve fibre loss, below the fifth percentile (P_5_). Consistent with this trend, no specific sectoral pattern of nerve fibre thinning was observed in the subgroup of eyes with the most severe loss of the pRNFLT or MRW. It remains unclear whether these differences between the data from the two study sites are related to different OCT technology and sectoral segregation, with potentially different sensitivity to nerve fibre loss, or whether there is a true difference in optic disc morphology.

Preservation of the temporal sector pRNFLT after preterm birth has been shown in several recent optic disc OCT studies (Fieß et al., 2017; Fieß, Schäffler, et al., 2022; Lehtonen et al., 2023; Wang et al., 2012a). It has been postulated that preterm birth is associated with a failure of multiple retinal layers to migrate away from the fovea, resulting in increased foveal thickness, and that these immature retinal layers could directly contribute to the temporal sector pRNFLT (Åkerblom et al., 2012; Wang et al., 2012b). In this study, we used the MDA index for characterization of foveal maturation (Bowl et al., 2018). The MDA index was abnormally low (below the fifth control percentile) in 38% of eyes in the VLBW group referring to thick fovea, with the highest incidence of 63% in the EP‐VLBW group of the HeSVA cohort. The MDA index, thus, proved to be a useful tool in this context to distinguish macular immaturity caused by preterm birth. However, the MDA index did not differ between the VLBW subgroups with different severity of optic disc OCT alterations, nor did it correlate with temporal sector pRNFLT. Hence, the optic disc OCT findings and foveal structure of these preterm‐born eyes are suggested to be parallel but independent reflections of neuroretinal development caused by birth with VLBW.

Many VLBW eyes showed thinning of the nerve fibres in the inferior sectors, which we should be aware of especially when evaluating the optic disc in suspected glaucoma. The MRW has been suggested to have better specificity to discriminate between normal and early glaucomatous optic disc than the pRNFLT (Yusof et al., 2022), and the pRNFLT/MRW ratio, in turn, to differentiate glaucoma from other optic neuropathies (Boussion et al., 2023). The pRNFLT/MRW ratio was similar in VLBW and control eyes of the HeSVA cohort, consistent with non‐glaucomatous optic disc alteration. The I/S pRNFLT ratio also remained normal in all VLBW eyes. Thus, the optic disc OCT indexes used in the differential diagnosis of glaucoma proved applicable also in this study of normotensive eyes, in which normal pRNFLT pattern had been violated by preterm birth with VLBW. However, the I/S MRW ratio was marginally lower in the VLBW group compared to controls, which may misdirect diagnostic evaluation. Another question is whether the loss of nerve fibres associated with preterm birth increases the outbreak risk of glaucoma. This remains to be seen by time as the first generations of previous preterm‐born VLBW populations get older.

The 17 VLBW participants born extremely preterm had a slightly larger optic disc size and thinner neural rim than both controls and VLBW participants born after 28 completed gestational weeks. Large optic disc size compared with controls has previously been reported in a photography‐based study in a Swedish preterm cohort born at 24–32 weeks' gestational age (Hellström et al., 1997) and in a German cohort of preterm born adults (age 18–50 years) (Fieß, Gißler, et al., 2022). A normal‐sized optic disc, but with reduced rim area and enlarged optic cup, has been found in children with periventricular leukomalacia, while reduced optic disc diameter, optic disc area and optic cup area have been reported in association with periventricular haemorrhage (Jacobson et al., 1997; McLoone et al., 2006). Both diagnoses are relatively common in VLBW born population. During the period when the two birth cohorts were born, brain MRI was not routinely included in the protocol of clinical evaluation. Thus, we do not know the incidence of subtle perinatal CNS changes and their potential impact on optic disc features in the studied VLBW participants. On the other hand, reduced rim area independent of evident neurological complications or ROP has previously been reported in a preterm‐born school‐aged cohort (Åkerblom et al., 2018). Apart from the differences in the optic disc size and neural rim, our data suggest the limited impact of extremely preterm birth per se on optic disc OCT. In addition to premature birth, retinal development is indirectly affected by many events during the perinatal phase, whereby the maturity of the retina does not solely correspond to the weeks of gestation.

All studied VLBW participants had ETDRS letter scores within the normal level (>80 letters) (Elflein et al., 2015). In contrast to a previous study by Wang et al. (2012a) reporting an association between temporal sector pRNFLT and visual acuity, we could not find such a correlation. Whether this is due to the bias in the study cohort including only participants with sufficient fixation (and visual acuity) for the OCT imaging remains to be questioned. On the other hand, normal visual acuity assessed by the high contrast ETDRS letter score is frequently seen in eyes with severely reduced neural rim and pRNFLT due to both congenital and acquired optic nerve diseases (Fisher et al., 2006).

The main strength of this study is the number of eyes evaluated and the representation of two different populations (Jussinniemi et al., 2024). The use of two different OCT platforms brought consistent findings in both study sites, which is a strength of the study, but it may also have caused differences in the results that could be solely related to the imaging technology. ROP stages have been shown to inversely correlate with nasal RNFL thickness (Park & Oh, 2015). Historical birth records were comprehensive but unfortunately did not include primary neonatal ROP findings or systematic brain imaging since such data were not obtained in the neonatal period. The potential impact of ROP or subtle CNS changes in the optic disc morphology therefore remains unclear in our study.

The VLBW participants included in the study were clinically healthy individuals with normal IOP. At ophthalmology clinics such a patient with a slightly abnormal appearance of the optic disc is relatively common and the question is how to distinguish normal variance or congenital alterations from acquired and potentially progressive diseases, such as glaucoma. In this context, also a history of preterm birth with VLBW should be considered.

## 5 CONCLUSIONS

Our results imply that birth history should be a routine part of medical history and also of adult patients undergoing evaluation of degenerative and acquired optic neuropathies later in life. It is important to recognize that changes in the optic disc OCT related to preterm birth with VLBW are common in clinically healthy individuals, also without previously treated ROP or diagnosed CNS abnormality. Further research on the association between foveal retina and optic disc morphology as well as retinal vasculature features is needed to better understand the long‐term effects of preterm birth on the ocular structures.

## Supporting information


Appendix S1.

Appendix S2.
