# Supplementary material for: Imprint of preterm birth with very low birth weight on optic disc OCT in adulthood—A two‐country birth cohort study
Source: Acta Ophthalmol. 2024 Oct 17;103(1):50–60. doi: 10.1111/aos.16771 (PMC11704842; doi:10.1111/aos.16771)
Supplement: Supplementary file 1 — Appendix S1. [file AOS-103-50-s002.docx]

| **Cerebral palsy (n=4)** |  |  |
| --- | --- | --- |
|  | Mean (SD) / n (%) | Range |
|  |  |  |
| **Perinatal data** |  |  |
| Gestational age (weeks) | 29.9 (2.1) | 27–32 |
| Extremely preterm birth (<28^th^ week) | 1 (25%) |  |
| Birth weight (g) | 1080 (120) | 900–1210 |
| Birth weight as SD score | -1,9 (2.1) | - 4.6–0.7 |
| Small for gestational age (SGA) | 3 (75 %) |  |
| Birth length (cm) | 37.5 (1.6) | 35–39 |
| Apgar score 1 min | 5.5 (3.3) | 2–9 |
| Apgar score 5 min | 5.5 (2.9) | 3–8 |
|  |  |  |
| **Parental data** |  |  |
| Maternal age (years) | 31.6 (3.7) | 28–37 |
|  |  |  |
| **Participant data** |  |  |
| Age at follow-up (years) | 37.9 (2.0) | 36.3–41.0 |
| Female | 2 (50 %) |  |
|  |  |  |
| **Eye data** |  |  |
| BCVA score, better eye | 92.8 (2.9) | 90–97 |
| BCVA score, worse eye | 87.3 (4.6) | 81–93 |
| Spherical equivalent (D) | -1.4 (1.9) | -5.25–0.125 |
| Intraocular pressure (mmHg) | 12.0 (2.4) | 10.0–16.0 |
|  |  |  |

**Supplementary Table S1a**. **VLBW with cerebral palsy.**

Background characteristics of VLBW participants with cerebral palsy in Helsinki Study of Very Low Birth Weight Adults.

Abbreviations: BCVA, best corrected visual acuity; D, diopter; VLBW, very low birth weight.
