# Supplementary material for: Imprint of preterm birth with very low birth weight on optic disc OCT in adulthood—A two‐country birth cohort study
Source: Acta Ophthalmol. 2024 Oct 17;103(1):50–60. doi: 10.1111/aos.16771 (PMC11704842; doi:10.1111/aos.16771)
Supplement: Supplementary file 2 — Appendix S2. [file AOS-103-50-s001.docx]

**Supplementary Table S1b. Optic disc morphology examined by optical coherence tomography (OCT).**

Number of examined eyes, OCT derived data for Bruch’s membrane opening (BMO), minimum rim width and peripapillary retinal nerve fiber layer thickness (pRNFLT) in different optic disc sectors and globally are presented as mean with standard deviations (SD) without adjustments. P values refer to comparisons between CP-VLBW and VLBW or control group, using mixed models with participant entered as a random effect and including adjustments for age, sex, and spherical equivalent of the refractive error for BMO area*, and additional adjustment with BMO for MRW and pRNFLT **.

Abbreviations: CP-VLBW, participants with diagnosed cerebral palsy and born with very low birth weight; HeSVA, Helsinki Study of Very Low Birth Weight Adults; VLBW, very low birth weight.
